# An In-Situ Fabrication Method of ZnO and Other Zn(II) Compounds Containing Polypropylene Composites

**DOI:** 10.3390/ijms24032357

**Published:** 2023-01-25

**Authors:** Katarzyna Kupińska, Maciej Michalik, Justyna Krajenta, Magda Bielicka, Karolina Halina Markiewicz, Beata Kalska-Szostko, Agnieszka Zofia Wilczewska

**Affiliations:** 1 Panamedica Maciej Michalik, Józefa Ignacego Kraszewskiego 18/7, 15-025 Białystok, Poland; 2 Doctoral School of Exact and Natural Sciences, University of Białystok, Ciołkowskiego 1K, 15-245 Białystok, Poland; 3 Faculty of Chemistry, University of Białystok, Ciołkowskiego 1K, 15-245 Białystok, Poland

**Keywords:** polypropylene composites, zinc oxide, antibacterial properties, nanoparticles

## Abstract

This study investigated the methods of preparation of zinc oxide-polypropylene nanocomposites and their antibacterial properties. Seven solutions with ZnO nanoparticles or zinc ions were formulated as a PP additive. Two methods of ZnO NPs syntheses were carried out: (1) a modified hydrothermal method where a water solution of zinc acetate dihydrate, PEI, and ammonia were mixed with a final pH 11; (2) a thermal decomposition of a water solution of zinc acetate in the presence of PEI and ammonia using a two-screw extruder. During the experiments, the influence of various amounts of particle stabilizer, heating of the solutions, and the temperatures of the syntheses were examined. As a result, the simultaneous crystallization of ZnO in the extrusion process confirmed this method’s attractiveness from the application point of view. Fabricated PP-ZnO composite shows antibacterial properties against *Staphylococcus aureus*, *Escherichia coli*, and *Klebsiella pneumoniae*.

## 1. Introduction

Nowadays, a big interest in polymer nanocomposites is being observed. Various polymers as a composite matrix as well as a wide range of metals or metal oxides of nano-size are being used. It is due to the potential of their synergic, unique physical, and chemical properties based on a large surface area to volume ratio of nanoparticles, and polymeric matrix features. High interfacial reactivity typical for individual nanoparticle properties is applied in many industrial branches of textile, medicine, packages, and many others. The most widely used nanoparticles (NPs) in polymer-based composites are silver, gold, titanium oxide, and zinc oxide [1,2,3,4,5].

Moreover, thanks to strong antibacterial properties, the ability of moisture and odor absorption, good thermal stability, and lack of irritation effect zinc oxide NPs are being used in wound dressing and various medical devices [6,7]. Some publications prove that zinc oxide NPs show antiviral activity against SARS-CoV-2 [8,9]. 

Various methods have been generally proposed to embed nanoparticles on textile and polymer surfaces. They can be classified into a one-step process (in-situ method) and a two-step process (ex-situ method) [10,11]. The in-situ process consists either of the immersion of synthetic fiber containing ZnO precursors into alkali media or the immersion of fiber or textile into zinc salt in alkali media [12]. The ex-situ synthesis of ZnO is divided into physical and chemical methods. The physical methods include electrospinning and melt spinning with fiber production [12]. The chemical application can be done by so-called layer-by-layer process or immersion of fiber into the dispersion of nano ZnO [10,11]. Homogeneous precipitation [13], wet chemical [14], and hydrothermal methods [15] are examples of the two-step processes which are being used more frequently than the one-step methods. They provide an approach to producing textiles immersed in ZnO solution. The superhydrophobic PP/ZnO nanocomposite surfaces were prepared through the solution casting method when PP granules were dissolved in xylene and then ZnO nanoparticles were added [16]. Becheri and co-workers [13] reported textile was immersed in ZnO solution [13,17,18]. Mao et al. [15] synthesized nano ZnO by the hydrothermal method and applied it to cotton fabrics. The textile was coated with SiO_2_ and then immersed in the solution of zinc nitrate and hexamethylenetetramine (HMT) at 80 °C for nano ZnO creation. Yadav and colleagues [18] prepared nanoparticles by the wet chemical method. The cotton fabric cut was immersed in the solution containing ZnO and acrylic binder then it was passed through a padding mangle before air drying. The hydrothermal method to synthesize ZnO nanowires on polyethylene fibers was described by Baruah et al. [19] with zinc nitrate hexahydrate and hexamethylenetetramine. Chemical bath deposition (CBD) was used by Fiedot and co-workers [20]. They modified the surface of the polyamide 6 (PA), polyethylene terephthalate (PET), and polypropylene (PP) using zinc oxide during the hydrothermal deposition in HMT and zinc nitrate. Anita et al. [21] prepared ZnO nanoparticles using soluble starch (stabilizing agent), zinc oxide nitrate, and sodium hydroxide (precursors). Then zinc oxide nanoparticles were microencapsulated and applied to a single jersey cotton fabric. Colmenares and co-worker [22] prepared composite filter materials by modification of commercially available polypropylene (PP) nonwovens with particles of nanorods of zinc oxide (ZnO/PP). Jakubiak and co-workers [23] used plasma discharge to improve the wettability and adhesion of ZnO nanoparticles. Electrospinning methods were used by Lee [24] and Kim [25]. The first paper described electrospun ZnO nanocomposite fibers from DMF solution for the development of UV-protective materials. Kim et al. [25] blended ZnO nanoparticles with a solution of nylon 6 to attain fine distribution of ZnO nanoparticles on/into the fibers. Teli et al. [26] showed a method when nanocomposites were melt-spun by adding nano ZnO loaded Linear Low-Density Polyethylene (LLDPE) masterbatch (MB) to the PET chips developed using nano ZnO during the spinning of PET composite fibers. Electrospinning can be also used for ZnO synthesis in a one-step process (in situ). Zhang et al. [27] obtained nanofiber by electrospinning PET solution containing zinc acetate dihydrate as a ZnO precursor. Montazer et al. [28] used the in-situ synthesis of ZnO on wool. For this process, the wool fabric was immersed in zinc acetate (basic condition) and kept at 90 °C for 1 h.

This study aimed to develop a new in-situ method, that allows obtaining a nanocomposite consisting of zinc compounds (including nano ZnO) in a polypropylene matrix as a one-step process. The polypropylene-based composite was obtained by modification of the extrusion process by doping of polymer with ZnO precursor as a water solution. This research concerns various precursor compositions allowing in-situ modification of the polymer and its study its physicochemical properties as well as antibacterial activity. 

Fabricated PP-ZnO composite can be afterwards converted to a useful product in post-treatment of modified polymer. 

## 2. Results

**Preparation of four zinc oxide precursor solutions.** (A) To the glass flasks with a water solution of zinc acetate dihydrate (ZnAc_2_) (27%), 6,84% of PEI branched was added. The mixture of liquid and solid substrates was subjected at room temperature to intensive stirring (500 rpm) for 3 h—Solution 1 (**S1**); (B) Solution **S2** was obtained by heating **S1** at 80 °C for 4 h; (C) Solution **S3** was obtained by dropwise addition of the ammonia solution up to pH 11. During the addition, a white precipitate of zinc hydroxide appeared, which dissolved before reaching the desired pH. The stirring was continued for another 3 h at room temperature; (D) Solution **S4** was prepared by heating **S3** at 80 °C for 4 h; (E) Solution **S5** was prepared according to the **S1** procedure with doubled amount of PEI. The differences in the fabrication process were presented in Table 1.

**Preparation of polypropylene doped with zinc compounds (E_1-4) in the extrusion process.**

**Figure 1:**
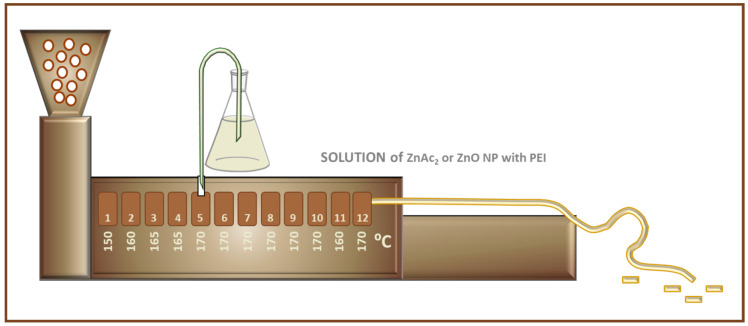
Schematic presentation of the extrusion set-up.

The extrusion process was carried out using a two-screw extruder and the following temperature setting in the subsequent heating zones: 

150/160/165/165/170/170/170/170/170/170/160/170 [°C]. A schematic presentation of the set-up is presented in Figure 1.

Before the extrusion process, the PP was subjected to a temperature of 90 °C for a purpose of material conditioning. Each solution was dosed into the 5th heating zone of the extruder’s cylinder by a peristaltic pump. The rate of the pump and the feeder were synchronized so that the final Zn concentration in granulate was around 2.5%. The extrudate was chilled in a water bath with a water temperature not higher than 30 °C. A masterbatches E_1, E_2, E_3, and E_4 for solution version S1, S2, S3, and S4, respectively were obtained. To improve the composite homogeneity and ZnAc_2_ decomposition efficiency, each masterbatch was extruded a second time at slightly higher temperatures [°C] at selected zones:

150/160/165/165/170/175/180/185/190/195/200/190 to obtain E_1x2, E_2x2, E_3x2, and E_4x2.

## 3. Results and Discussion

### 3.1. ZnO Precursor Solutions Preparation

Four different methods of zinc oxide precursor preparation were performed (for details see Table 1). The obtained samples were then examined in liquid or dry form by various physicochemical techniques. The results of the selected analyses are presented in the following paragraphs.

#### 3.1.1. Electron Microscopy—Morphology Determination

**Figure 2:**
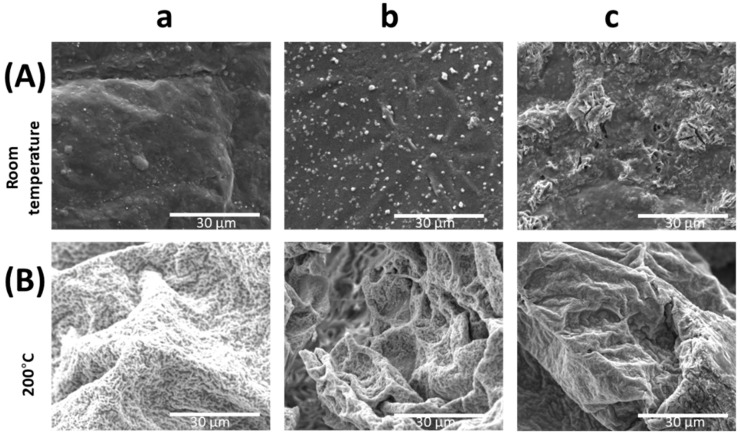
SEM images of samples dried at room temperature (top row—A) and 200 °C (bottom row—B); S1 (**a**), S3 (**b**), S4 (**c**).

A small amount of solutions were drop cast and dried at room temperature on SEM Al holders. For comparison, samples dried at 200 °C were prepared. The received samples were characterized and presented in Figure 1 and Figure 2.

**Figure 3:**
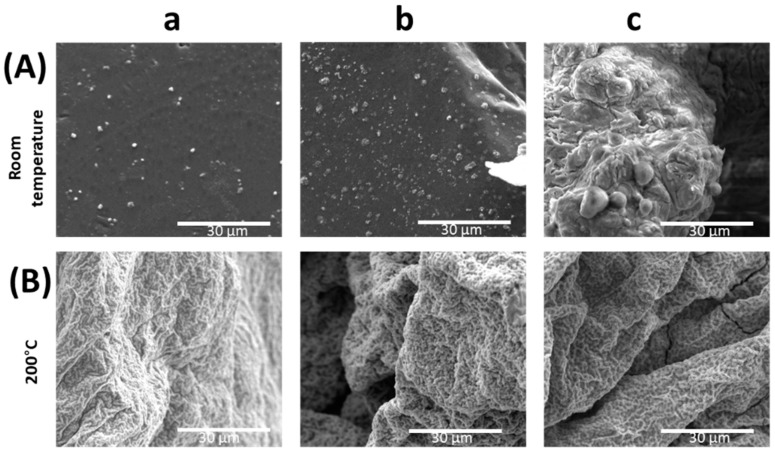
SEM images of samples dried at room temperature (top row—A) and 200 °C (bottom row—B); S5 (**a**), S6 (**b**), S7 (**c**).

On the SEM images (Figure 2 and Figure 3) a similar pattern of synthesis progression was observed, although solutions were treated in various temperature conditions. When samples were dried at room temperature some crystals appeared (Figure 2a and Figure 3a) visible as light spots on the surface. Therefore, forms of precipitated ZnO or its byproducts that arise as an effect of basic PEI presence, and sample dehydration were observed. As the ammonia solution was added (more basic solution)—Figure 2b and Figure 3b—the number of crystals increased. The size of the particles is much smaller in the case of the lower amount of PEI. In the images of the solutions dried at elevated temperatures bigger, irregular forms are visible. The samples dried at 200 °C look very similar to each other in all sample cases. However, no significant differences in crystallite size were observed suggesting good homogeneity of the film. 

**Figure 4:**
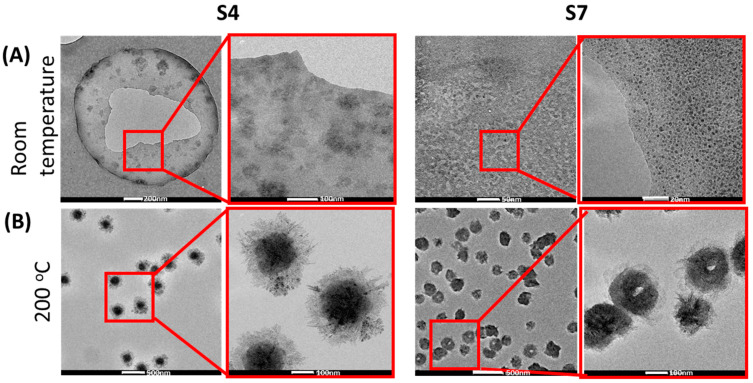
TEM images of samples S4 and S7 dried at room temperature (**A**) and 200 °C (**B**) in lower and higher magnification.

Additionally, for nanoscale object visualization, TEM observation of samples prepared in presence of ammonia, and heated for 4 h at 80 °C dried at RT (S4 in Figure 4) was performed. For comparison, to mimic the high-temperature treatment—as in the extruder, samples were dried at 200 °C (S7 in Figure 4). In Figure 4A very small particles 7 ± 3 nm (S7) or 38 ± 7 nm (S4) are seen. Their size depends on the amount of PEI precursor applied. After heat treatment of 200 °C (Figure 4B) a characteristic shape of balls or nests is visible of the size 170 ± 20 nm, or 220 ± 50 nm for S4 and S7, respectively. The shape and the size depends on the amount of ZnO precursor used.

The microscopic observation proved the influence of elevated temperature on the growth of particles. Room temperature drying results in the precipitation of very small objects (below 10 nm). Elevation of temperature causes structure growth. Therefore, the extrusion process is expected to favor particle growth more than the initiation of seed creation as well. Those phenomena are known from other nanoscale systems [29,30,31].

#### 3.1.2. X-ray Diffractions—Structural Characterization

**Figure 5:**
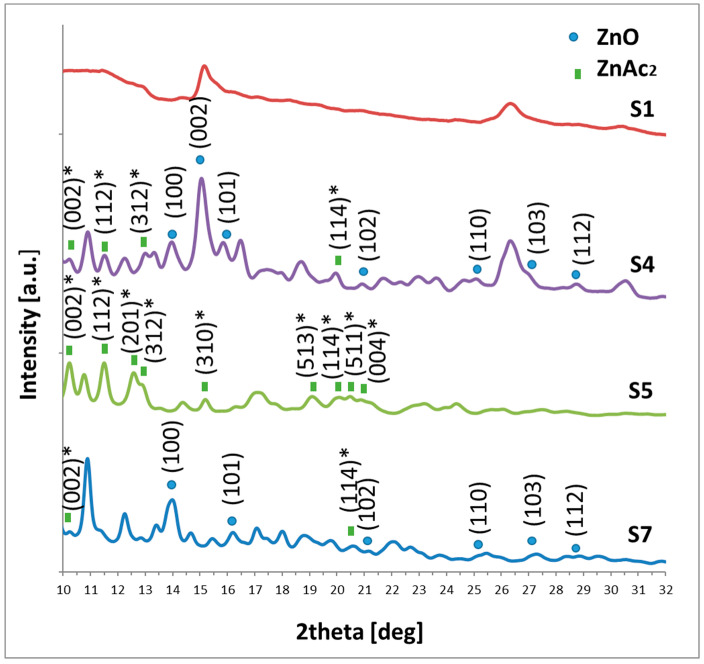
Diffraction results for samples: S1, S4, S5, and S7. () Miller indexes of ZnO, ()* Miller indexes of ZnAc_2_.

To monitor the influence of preparation conditions on ZnO crystallization X-ray diffraction measurements were taken for samples S1, S4, S5, and S7. Such studies allow confirmation of whether composites contain ZnO in their structure. Obtained diffractograms are collected in Figure 5. By dots are assigned ZnO patterns which correspond to positions of peaks indexed as (100), (002), and (102) in Miller nomenclature of ZnO crystals [32].

According to the diffraction data (Figure 5) only in the S4 and S7 samples, a trace of ZnO forms appeared. Other diffractograms are dominated by zinc acetate peaks, other zinc compounds, or amorphous phases [33]. These results suggest that the heat treatment causes the decomposition of ZnAc_2_ and its transformation to ZnO and no other stimuli are needed [34]. Such observation allows concluding that the proposed solution can be an effective precursor for PP-ZnO composite fabrication.

### 3.2. Extrusion—Technical Approach

For PP-ZnO composite preparation solutions S1, S2, S3, and S4 were used. The extrusion process was carried out using a two-screw extruder with the following temperature setting at each section of the cylinder [°C]:

150/160/165/165/170/170/170/170/170/170/160/170. Solutions S1, S2, S3, and S4 were dosed into the 5th heating zone of the extruder’s cylinder by a peristaltic pump. The rate of the pump and the feeder were synchronized so that the final Zn concentration in granulate was around 2.5%. A masterbatches E_1, E_2, E_3, and E_4 for solutions S1, S2, S3, and S4, respectively, were obtained. After taking a sample, each masterbatch was extruded a second time at slightly higher temperatures at selected zones [°C]:

150/160/165/165/170/175/180/185/190/195/200/190) for better ZnO particle distribution (Ex2). It is observed that double extruded extrudate had darker color, probably due to partial polymer degradation. 

#### 3.2.1. Melt Flow Index—Mechanical Properties

**Figure 6:**
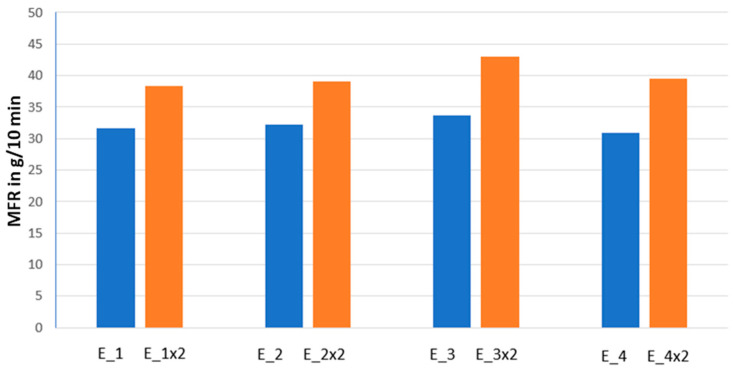
Influence of the second extrusion on the MFR value.

The Melt Flow Index of obtained granulates was performed after each step of the process. The results of the experiments are shown in Figure 6. For the determination of the influence of extrusion on MFR value results are presented pairwise (first and the second extrusion of the same sample).

The MFR value of the masterbatch extruded once stays in a range: of 30.95–33.64 g/10 min. For the second extrusion, the temperatures were set slightly higher. The composites obtained in this process have therefore higher MFR which stays in the range of 38.36–43.02 g/10 min which ensures its mechanical properties are in an acceptable range. Therefore, the following process is possible. 

#### 3.2.2. Optical Microscopy—Composite Morphology

**Figure 7:**
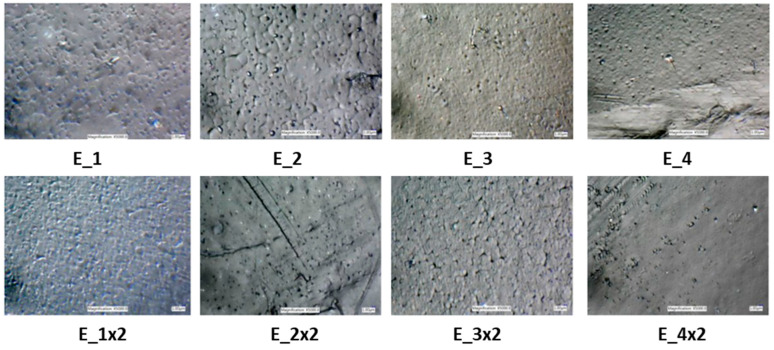
Optical microscope images (magnification ×5000) of one-time and two-times extruded samples.

The morphology of the granulates using a high-resolution optical microscope under magnification ×5000 was observed (Figure 7). It is seen that samples E_2x2 and E_4x2 (no ammonia, after the second extrusion) are smoother. Extrudates E_1, E_1x2, E_3, and E_3x2 look very similar. Their surface is flat and only some tiny, evenly dispersed particles are observed which affirms the uniform distribution of additives in a polymeric matrix. 

#### 3.2.3. Zn Content Determination in Granulates

The E_1, E_1x2, E_2, and E_2x2 granules were tested for the total amount of zinc by flame atomic absorption spectroscopy (FAAS). The results are listed in Table 2.

According to the FAAS results, the amount of zinc in the composites is slightly higher than the nominal value which can be related to the relative mechanical movement of polymer and solution in the dispensers. The zinc content in the granulates extruded twice is slightly lower due to the elution of surface-bonded zinc compounds during the extrudate cooling in a water bath. This observation is in agreement with the fabrication procedure and proves that Zn compounds are evenly spaced in the polymer matrix.

#### 3.2.4. Thermal Analysis

**Figure 8:**
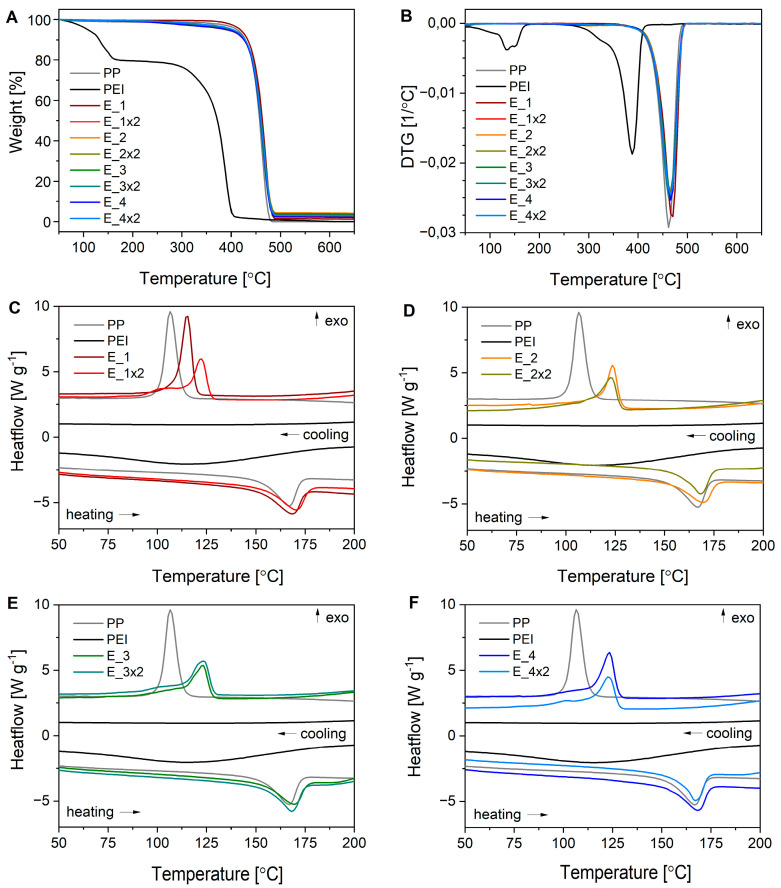
The TGA (**A**), DTG (**B**), and DSC (**C**–**F**) results for PP, PEI, and composites E_1, E_1x2, E_2, E_2x2, E_3, E_3x2, E_4, and E_4x2.

The thermal stability and decomposition of PP, PEI, and composites E_1, E_1x2, E_2, E_2x2, E_3, E_3x2, E_4, and E_4x2 were studied using thermogravimetric analysis. The TG and DTG curves of the tested materials are presented in Figure 8A,B. A weight loss in the temperature range up to 150 °C observed for PEI is most likely due to the removal of water. The degradation of PEI starts at 250 °C, whereas PP is thermally stable up to 400 °C. The composites start to degrade above 250 °C; however, the observed weight losses are slight up to 400 °C, suggesting the presence of small amounts of PEI. PP and PEI decompose totally in the applied temperature range, whereas in the cases of composites residues at the level of 1–4% are observed (Figure 8A, Table 3). These residues are associated with the presence of zinc and/or zinc compounds (i.e., zinc oxide), which do not decompose in the tested temperature range. 

The temperatures of phase transitions and crystallinity of composites were determined based on DSC experiments (Figure 8C–F) and are collected in Table 3. The addition of PEI and Zn compounds only slightly affected the melting temperature and crystallinity of composites compared to the starting PP. On the other hand, it significantly changed their crystallization behavior, generally, the width and maximum of the crystallization peak increased. 

### 3.3. Bacteriostatic and Antibacterial Investigations

The 0.5 mm films were made by press molding out of the E_1, E_1x2, E_2, E_2x2, E_3, E_3x2, E_4, and E_4x2 samples and bacteriostatic testing according to ISO 20645 was performed. Tested materials were placed on agar plates seeded with *E. coli* or *S. aureus* bacteria. As a reference sample, a polypropylene film without additives was used. Therefore, prepared Petri dishes were incubated at 44 °C and 37 °C for *E. coli* and *S. aureus*, respectively. A lack of *S. aureus* inhibition growth was observed in each sample. The film of E_2 composite was clean from *E. coli*. Partial inhibition of *E. coli* growth was observed for samples E_1 and E_3. The films prepared out of composite extruded twice show no bacteriostatic effect. 

An antibacterial activity test of E_1, E_1x2, E_2, E_2x2, E_3, E_3x2, E_4, and E_4x2 films against *Staphylococcus aureus* ATCC 6538, *Escherichia coli* ATCC 11229 and *Klebsiella pneumoniae* ATCC 4352 according to ISO 20743 was performed. A polypropylene film without additives was used as a reference sample (Table 4). 

According to ISO 20743, a result value between 2 < A < 3 means significant bacterial activity and A ≥ 3-strong. The best results were obtained for sample E_1, which contained a solution prepared without heating and without ammonia solution. A slightly lower antibacterial activity index shows samples E_2 and E_3 (solution prepared with heating but without ammonia, and without heating with ammonia, respectively). 

The presented results demonstrate that the bacteriostatic and antibacterial activity of fabricated PP-ZnO composites strongly depends on the precursor used and its fabrication history. Therefore, a detailed analysis of this dependence needs more investigation.

## 4. Materials and Methods

Zinc acetate dihydrate and ammonia solution (30%, *w*/*w*) was supplied by Chempur. Polyethyleneimine (PEI) branched was purchased from Sigma-Aldrich. Polypropylene fiber grade (Moplen HP561R, MFR = 25 g/10 min) was obtained from LyondellBasell. Water was deionized by a Polwater deionizator.

**Scanning electron microscopy** (SEM) and transmission electron microscopy (TEM) images were done to determine the morphology and size of the ZnO particles in the zinc precursor solutions and polymeric composites, respectively. SEM and TEM were performed using—a scanning electron microscope (INSPEC 60) integrated with Energy Dispersive X-ray (EDX) and FEI Tecnai G2 X-TWIN 200 kV microscope, respectively. To prepare samples for imaging a drop of the solutions S4 or S5 were placed on SEM holders or TEM Cu 400 mesh grids and dried at room temperature or 200 °C on the external heating plate until solvent evaporation.

**Optical Microscope**. The modified polymeric base in form of granules (after the extrusion process) cross-section was observed under the high-resolution optical microscope (Keyence VHX 6000) to determine the morphology of the composite.

**X-ray diffraction** (XRD) was performed using Agilent Technologies SuperNova diffractometer with a Mo micro-focused source (K_α2_ = 0.713067) device for ZnO fraction identification. For this purpose, dried solution residues were placed in a goniometer on a nylon loop via highly viscous oil.

**Melt Flow Index** (MFR) values of the obtained masterbatches were determined on Dynisco LMI plastometer with 2.16 kg load and 230 °C. The procedure was carried out according to ISO 1133-1.

**Thermogravimetric analyses** (TGA) were performed using a Mettler Toledo Star TGA/DSC unit. Samples weighing 2–3 mg were placed in aluminum oxide crucibles and heated from 50 °C to 900 °C at 10 °C min^−1^ under an argon flow rate of 40 mL min^−1^. An empty pan was used as the reference. 

**Differential scanning calorimetry** (DSC) measurements were performed using a Mettler Toledo Star DSC unit. Samples weighing 2–3 mg were placed in an aluminum crucible, and an empty pan was used as the reference. The samples were measured using the following procedure: heated from 0 to 230 °C at a rate of 10 °C min^−1^, held isothermally for 3 min, and then cooled to 0 °C at a rate of −30 °C min^−1^. For each sample, two heating/cooling cycles under an argon flow rate of 200 mL min^−1^ were performed. Characteristic temperatures (melting and crystallization), and degrees of crystallinity were determined using Mettler Toledo STARe software based on the second cycle of heating and cooling. The heat of melting Δh100% = 207.10 J g^−1^ was assumed for 100% crystalline PP [35].

**Atomic Absorption Spectroscopy (AAS)**. The total amount of zinc in the samples was examined by Atomic Absorption Spectroscopy (AAS) Solaar M6 equipped with a flame atomizer (Thermo Electron Corporation, Loughborough, UK) and zinc hollow cathode lamp (SMI-LabHub Ltd., Gloucester, UK). After the sample mineralization, the absorption of the obtained solutions was registered at 213.859 nm. The measurements were taken at the fuel flow rate of 1.4 L/min and the burner high of 8.6 mm. During the analysis, a deuterium lamp was used for background correction.

**Bacteriostatic activity.** The E_1-4 composites were tested for bacteriostatic activity according to ISO 20645. 0.5 mm films were made by press molding out of the obtained masterbatch. Tested materials were placed on agar plates seeded with *Staphylococcus aureus* or *Escherichia coli* bacteria. As a reference sample, a polypropylene film without additives was used. Therefore, prepared Petri dishes were incubated at 44 °C and 37 °C for *E. coli* and *S. aureus*, respectively.

**Antibacterial activity** test against *Staphylococcus aureus* ATCC 6538, *Escherichia coli* ATCC 11229, and *Klebsiella pneumoniae* ATCC 4352 according to ISO 20743 was performed for the films obtained from the masterbatches. A polypropylene film without additives was used as a reference sample. 

## 5. Conclusions

For the first time, the ZnO-modified polypropylene was obtained via extrusion at elevated temperature from a water solution containing ZnAc_2_ precursor stabilized by polyimine. Fabricated material exhibits comparable mechanical properties to pure (non-modified) PVP and parallel antibacterial properties against *Staphylococcus aureus* ATCC 6538, *Escherichia coli* ATCC 11229, and *Klebsiella pneumoniae* ATCC 4352 thanks to the presence of ZnO or other Zn compounds. 

Previously carried out modifications of textile materials with nanoparticles of zinc compounds consisted mainly of surface alterations of already formed polymer fibers. This was mainly carried out using the dipping method. 

The method presented in this work is unique due to: (a) the transformation of zinc compounds into nanoparticle structures directly during the extrusion process; (b) the volumetric rather than surface introduction of zinc compounds into the polymer; (c) the use of aqueous solutions of particle precursors and their introduction into the nonpolar polymer; (d) the reduction of the amount of nanoparticles of zinc compounds throughout the process, by forming them directly in the polymer structure; (e) the simplification of the method for the formation of polymer-nanoparticle composites—one-step process; (f) the uniform distribution of nanoparticles in the polymer; (g) the obtaining of the PP-ZnO composite in granular form, which can then be easily converted into a useful product. 

## Figures and Tables

**Table 1:** Experimental data of ZnO precursor solutions preparation.

Sample Name	% of PEI	pH	Reaction Temperature °C
**S1**	6.84	-	RT
**S2**	6.84	-	80
**S3**	6.84	11	RT
**S4**	6.84	11	80
**S5**	13.68	-	RT
**S6**	13.68	11	RT
**S7**	13.68	11	80

**Table 2:** Zn content measured by FAAS method.

Sample	Nominal Zn Content [%]	Experimental Zn Content [%, *w*/*w*]
**E_1**	2.5	2.55
**E_1x2**	2.5	1.98
**E_2**	2.5	2.79
**E_2x2**	2.5	2.71

**Table 3:** Thermal properties of PP, PEI, and composites E_1, E_1x2, E_2, E_2x2, E_3, E_3x2, E_4, and E_4x2. *T_m_*, melting temperature; *T_c_*, crystallization temperature; *C*, degree of crystallinity.

Sample	Residue at 650 °C Measured by TG [%]	*T*_m_ [°C]	*T*_c_ [°C]	*C* [%]
**PP**	0	166.2	107.6	43
**PEI**	0	-	-	-
**E_1**	1.2	167.8	115.7	40
**E_1x2**	2.3	169.7	122.6	41
**E_2**	4.1	168.9	124.5	38
**E_2x2**	3.5	167.5	123.4	40
**E_3**	3.6	168.8	123.4	42
**E_3x2**	2.1	167.4	124.0	46
**E_4**	2.0	167.8	124.0	41
**E_4x2**	2.9	166.6	123.4	38

**Table 4:** Antibacterial activity testing results according to ISO 20743.

Sample	Antibacterial Activity A		
	*Staphylococcus Aureus* ATCC 6538	*Escherichia coli * ATCC 11229	*Klebsiella pneumoniae * ATCC 4352
**PP**	0.00	0.00	0.00
**E_1**	7.62	7.23	3.54

## Data Availability

The raw/processed data required to reproduce these findings cannot be shared at this time as the data also forms part of an ongoing study.
